# Tuberculosis Epidemiology at the Country Scale: Self-Limiting Process and the HIV Effects

**DOI:** 10.1371/journal.pone.0153710

**Published:** 2016-04-19

**Authors:** Felipe Augusto Maurin Krsulovic, Mauricio Lima

**Affiliations:** 1 Pontificia Universidad Católica de Chile, Santiago, Chile; 2 Pontificia Universidad Católica de Chile, Santiago, Chile; Fundació Institut d’Investigació en Ciències de la Salut Germans Trias i Pujol, Universitat Autònoma de Barcelona, SPAIN

## Abstract

**Background:**

The global spread of the human immunodeficiency virus (HIV) is the main hypothesis behind tuberculosis (TB) positive trends in the last decades, according to modeling studies and World Health Organization Reports (WHO). On one hand, TB (WHO) reports do not explicitly consider a modeling approach, but cover country and global TB trends. On the other hand, modeling studies usually do not cover the scale of WHO reports, because of the amount of parameters estimated to describe TB natural history. Here we combined these two principal sources of TB studies covering TB High Burden Countries (HBCs) dynamics. Our main goals were: (i) to detect the endogenous component of TB dynamics since 1974 for TB HBCs; and (ii) to explore the HIV exogenous effects on TB models`parameters.

**Methods and Findings:**

We explored the relationship between the TB per capita population rate of change (*R*_*I*_) and the infectious class size following an endogenous/exogenous framework. *R*_*I*_ can be affected by intra-population processes (i.e. competition, predation) and exogenous variables like HIV. We found that TB dynamics had always a strong endogenous component, represented by a negative correlation between TB population size and *R*_*I*_, which was captured by the discrete logistic model. Moreover, we explored the HIV exogenous effects on TB models`parameters. We found that overall the TB+HIV logistic model was more parsimonious than TB model alone, principally in the African region. Our results showed that HIV affected principally TB carrying capacity, as expected by the known HIV effects on TB natural-history. We also tested if DOTS (Directly Observed Treatment Short-Course Strategy), poverty levels and BCG (Bacillus Calmette-Guérin) coverage explained the models´ residuals variances, but they did not.

**Conclusions:**

Since 1974, TB dynamics were categorized in distinct chronological domains, with different dynamics but nearly the same underlying mechanism: a negative relationship between *R*_*I*_ and infected class size (i.e. self-limiting). In the last decades, not only HIV spread represented a new TB chronological domain, but it also has been pushing TB carrying capacity (*K*) to higher levels. TB has a complex natural-history and imposes real challenges to model its dynamics. Yet, we were able to explore and reveal the main drivers of TB dynamics for HBCs since 1974, through a simple approach. Based on our results, we suggest that the endogenous view should be considered as a plausible hypothesis to model and explain TB dynamics and that future World Health Organization reports could include the endogenous/exogenous framework as a supplement to help to decipher the main drivers of TB dynamics and other diseases.

## Introduction

Tuberculosis (TB) is an ancient air-borne disease [1], with high incidence and mortality rates among human populations [2–4]. Despite the advances as BCG vaccine (Bacillus Calmette-Guérin) and antibiotics, TB started to rise again, mainly in low income countries. Although the appearance of multi-drug resistance TB strains may have also played a role, the global spread of the human immunodeficiency virus (HIV) has been the main driver of recent TB positive trends, especially in Asia and Africa [3–7].

The HIV and TB levels peaked in the 1990s and several countries joined in a global effort to reduce TB burden. The programs Directly Observed Treatment Short-Course (DOTS) strategy, the Stop TB strategy and TB-related Millennium Development Goals established general goals aiming to reduce TB levels by half until 2015. Some countries are closer to these goals than others and a major challenge seems to be the link between HIV and TB, principally in Africa where the level of co-infections can reach 75% of TB patients [4].

The knowledge about TB burden comes from two key sources of information: modeling studies and TB World Health Organization reports (WHO). The usual approach to model TB diseases is the SEIR (Susceptible->Expose->Infect->Recover) type models [8]. Due to its inherent variable natural-history, authors have been modeling TB dynamics with distinct assumptions and types of models that range from unstructured ordinary differential equations to spatially structured models, with many compartments [8–21] (S1 Text). WHO reports usually present global and country scale TB trends, which are avoided by modeling studies, because of the amount of SEIR parameters needed to be estimated to capture TB dynamics. Here we hope to combine these two principal sources of TB studies, by exploring the TB High Burden Countries dynamics from 1974 until 2012, using simple population dynamic models. Population ecologists have developed a simple framework using principles adopted from system dynamics and biological interactions, which helps to explore the contributions of endogenous and exogenous processes even in potential complex systems. Briefly, density-dependency (endogenous process) occurs when the state of a variable (i.e. population size) is determined, positively or negatively, by its own previous states. Density–independency (exogenous process) occurs when a variable affect another but is not affected by the changes it causes. There is no feedback structure between them [22–30]. Climate variables and governmental disease control policies are known examples of exogenous pulse/press perturbations regarding diseases [22]. Both processes may operate to determine the dynamic behavior of a particular population through time. Therefore, a more complete understanding of the dynamics of a population is achieved when both endogenous and exogenous processes are taken into account.

It is widely known that HIV may affect TB transmission rate, re-infection risk, the herd immunity effect of BCG vaccine and progression rates from susceptible to infected class [12, 13, 19, 20]. Hence, as suggested in WHO reports and HIV-TB studies, HIV may be acting as a positive press exogenous force on TB dynamics. This study had two general goals: (i) to detect the endogenous component for TB dynamics since 1974 for TB Higher Burden Countries (HBCs); and (ii) to explore and the HIV exogenous effects on TB model parameters. We also explored the effects of other variables that could affect TB levels mentioned in the WHO reports, including poverty levels, vaccination coverage and DOTS (see below).

## Materials and Methods

The complex TB natural-history would require the estimation of a large amount of parameters for deciphering TB dynamics for the HBCs following the SEIR approach (S1 Text). Hence, we deviated from the usual SEIR type models. Here we focused only on TB annually reported cases (i.e. diagnosed, new and relapsed) aiming to reveal general patterns of TB infectious class dynamics. Annual TB reported cases were gathered from WHO reports available at WHO website (www.who.int/en/).

The realized per capita population rate of change is the central stone of the framework used here and is estimated as:

$$R_{I}=Ln\left(I_{t}/I_{t-1}\right)$$ (1)

where *R*_*I*_ is the realized rate of change of the average TB patient in the infectious class over a time interval and *I*_*t*_ and *I*_*t* − 1_ are the number of annual new and relapse infectious cases at consecutive years.

*R*_*I*_ can be affected by intra-population processes (including self-limiting and predator-prey interactions) and exogenous variables, including HIV (22–30). Based on TB literature, system dynamics principles and disease dynamics theory, the *R*_*I*_ of TB infectious class can be expressed as:

$$R_{I}=f(I_{t-d},DOTS_{t-1},P_{t-1},BCG_{t-1},HIV_{t-1}{}_{,},V)$$ (2)

where *R*_*I*_ is an unidentified function (*f*) of the population endogenous processes (*I*_*t* − *d*_), exogenous variables (*DOTS*_*t* – 1_, *P*_*t* – 1_, *BCG*_*t* – 1_, *HIV*_*t* – 1_) and other variables (*V*) that may also determine the environment where TB dynamics takes place.

Assuming a constant environment (exogenous variables), a negative relationship between *R*_*I*_ and *I*_*t* − 1_ (i.e. *d* = 1) suggests that as the infected portion of the population increases, infected individuals face more difficulties to recruit susceptible individuals, reducing the realized per capita rate of transmission (22). This process resembles the law of diminishing returns (i.e. self-limiting), which has the intra-specific competition as its ecological analogous. It represents a negative first-order feedback between *R*_*I*_ and *I*_*t* − 1_. A negative relationship between *R*_*I*_ and *I*_*t* − 2_ suggests that TB population size affects itself with a delay, probably through another variable (i.e. second-order feedback) as the susceptible class, a predator-prey interaction. In this case, a circular behavior is observed between *R*_*I*_ and *I*_*t* − 1_. *R*_*I*_ can also be a positive function of *I*_*t* − 1_. Population size may enhance individual probabilities of finding resources and refuges, which is the “Allee effect”. Populations under this phenomenon describe a positive correlation between *R*_*I*_ and population size. Also, *R*_*I*_ may be independent of the infectious population size. Under a constant environment, population may increase unbounded at constant R levels [22–29].

Since data visual inspection revealed abrupt changes in TB dynamics since 1974, it was necessary to cut TB dynamics in distinct chronological periods. Although the cut procedure is subjective, it is based on data behavior (TB and *R*_*I*_ levels and trends). Briefly, if *R*_*I*_ was declining and suddenly increased and then started to decline again, we assumed that the time series should be cut prior to the increase and the periods should be analyzed separately (S3 Text). Despite distinct cut-points may produce distinct results, it would probably not change our general findings, principally because we focused on TB last period of growth (see below).

The TB chronological periods and positive trends imposed a challenge to select the model to fit the data. Model selection was based on the visual inspection of relationship between *R*_*I*_ and *I*_*t* − 1_. Population Dynamic Theory states that: *R*_*I*_ should increase with population size when some kind of cooperation exists and affects reproduction and survival and that *R*_*I*_ should decline with population size when an intra-specific competition is operating and affecting negatively the reproduction and positively mortality [25]. Whenever a decrease or increase in *R*_*I*_ with *I*_*t* − 1_ was observed, we fitted the following discrete models, respectively:

$$R_{I}=R_{\max}\left(1-{\left(\frac{I_{t-1}}{K}\right)}^{Q}\right)$$ (3)

$$R_{I}=R_{\max}{\left(\left(\frac{I_{t-1}}{E}\right)-1\right)}^{T}$$ (4)

where *R*_max_ is the maximum per capita rate of change, in the previous year, *K* is the stable population size at equilibrium (carrying capacity) and *Q* is a non-linear term that reflects the intensity of intra-specific competition around *K*. *E* is an unstable equilibrium population size for the discrete Allee model (Eq 4), where, above it, population always grows and below it, population tends to extinction. *T* represents how fast population deviates from the unstable equilibrium size [25, 31].

Most countries exhibited a negative relationship between *R*_*I*_ and *I*_*t* − 1_, suggesting the logistic model (Eq 3) as the first candidate to model and capture TB dynamics [22]. Nevertheless, two countries showed an increase followed by a decrease in *R*_*I*_ levels (UR Tanzania and Bangladesh. See below). In these cases we fitted the following Allee-Logistic model:

$$R_{I}=R_{\max}\left(\left(\frac{I_{t-1}}{E}\right)-1\right)*\left(1-\left(\frac{I_{t-1}}{K}\right)\right)$$ (5)

where most parameters are the same as in Eq 3 and Eq 4, except for the absence of the *Q* and *T*. In this formula, we assumed that *R*_*I*_ behaved as a quadratic function of the *I*_*t* − 1_.

The *R*_*I*_-function (Eq 2) shows that *R*_*I*_ can also be affected by exogenous variables like the DOTS (*DOTS*_*t* − 1_), poverty levels (*P*_*t* − 1_), BCG coverage (*BCG*_*t* − 1_), HIV (*HIV*_*t* − 1_) and other variables (*V*) which may also determine the environment where TB dynamics takes place. Here, we were specifically interested in exploring and estimating the HIV impact in TB dynamics. As already mentioned, most countries showed periods of logistic growth, and because the logistic model has simple biological interpretations, we can explore the HIV effects on TB model parameters. We took the last period of growth of TB time series for each country and tested three types of HIV exogenous effects [24]: Lateral effect, where HIV affects only TB carrying capacity (Eq 6); Additive effect, when HIV affects both *R*_max_ and *K* (Eq 7); and Non-linear effect, where HIV affects the *Q* parameter (Eq 8).

$$R_{I}=R_{\max}\left(1-{\left(\frac{I_{t-1}}{K+gHIV_{t}}\right)}^{Q}\right)$$ (6)

$$R_{I}=R_{\max}\left(1-{\left(\frac{I_{t-1}}{K}\right)}^{Q}+gHIV_{t}\right)$$ (7)

$$R_{I}=R_{\max}\left(1-{\left(\frac{I_{t-1}}{K}\right)}^{Q+gHIV_{t}}\right)$$ (8)

where *g* is the linear coefficient that measures the effect of HIV on TB model parameters.

We tested HIV effects on TB time series for distinct time lags (*t*) to obtain the best model, which was selected based on the coefficient of determination (R^2^) and the Akaike Information Criterion (AIC). Models with higher R^2^ and lower AICs are more parsimonious and hence, were selected. We FItted Eqs 3–8 using the nls library in R using non-linear regression analysis [32]. We used the coefficient of determination (R^2^) from the above TB models (without HIV) as an estimation of the contribution of the endogenous processes to TB dynamics.

Due to the low degrees of freedom for the last periods of TB growth (see below), it was not always possible to properly estimate the additive (Eq 7) and non-linear HIV effects (Eq 8). Hence, we took the residuals from TB logistic model without HIV (Eq 3) and TB+HIV lateral model (Eq 6) and explored whether the non-HIV variables (DOTS, poverty levels and BCG coverage) individually explained the remaining variance, with Pearson´s product moment correlation coefficient. Detection of TB cases (DT) and treatment success (TS) are two key components of the DOTS strategy. WHO adopted a 70% detection rate and treatment success of 85%, expecting that these levels would lead to a 5% year reduction in the TB incidence rates. We collected from WHO web page the annual countries percentage of TB detected cases (DT) by sputum-smear microscopy and the percentage of these cases successfully treated (TS) From 1974 and 2012, DT and TS for most of all HBCs were below the WHO levels [4, 5]. We used per capita GDP as a proxy of poverty levels. Per capita GDP was estimated by the ratio between country GDP and human population size obtained from the World Bank web site (www.worldbank.org). BCG coverage and HIV reported cases were obtained from Gapminder web site (www.gapminder.org/data/). DT and TS time series usually were available from 1990 on, and HIV and BCG coverage since 1980.

In the main text, we present the TB dynamics and analyses for South Africa, Nigeria, Kenya, Mozambique, UR Tanzania, Zimbabwe, Brazil, Bangladesh, Cambodia and China, as a sample of TB dynamic behaviors and statistical results observed for the HBCs countries. For a complete appreciation of the results found in this study, see the supplementary material (S1 Table, S1 and S2 Figs).

## Results

**Fig 1:**
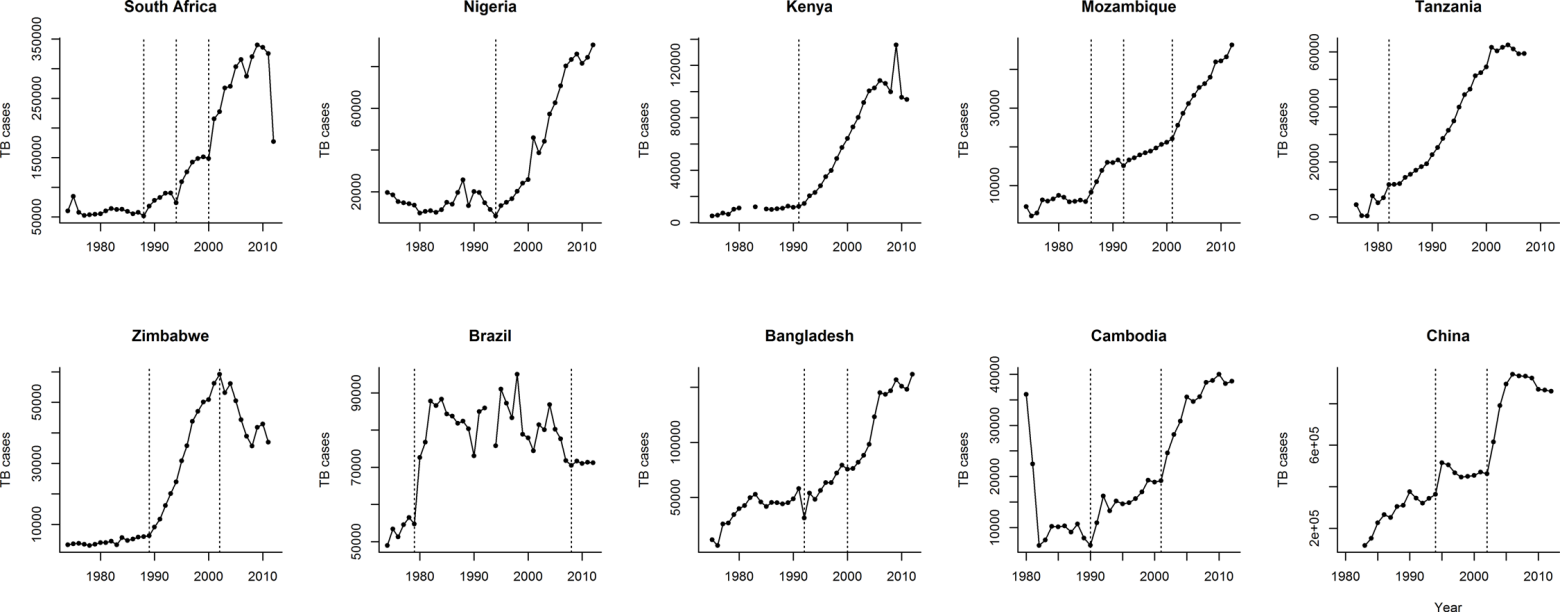
TB time series of South Africa, Kenya, Mozambique, UR Tanzania, Zimbabwe, Brazil, Bangladesh, Cambodia and China since 1974. Dashed vertical lines refer to the years where time series suffered dynamic changes.

**Fig 2:**
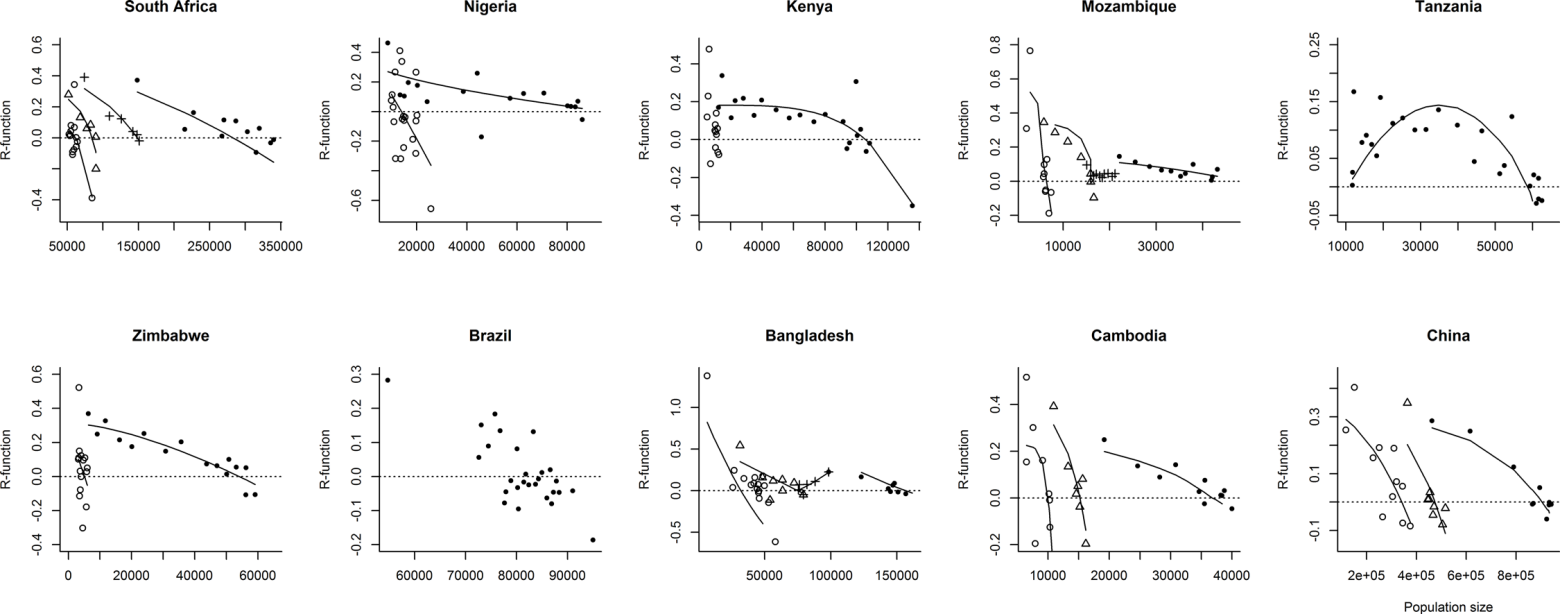
*R*_*I*_-functions for each country and TB periods of growth for South Africa, Kenya, Mozambique, UR Tanzania, Zimbabwe, Brazil, Bangladesh, Cambodia and China. The symbols refer to the chronological *R*_*I*_ periods: -○- the first, -Δ- the second, -+- the third and -●- the fourth. Only significant fits for the discrete models are shown with regression lines. *R*_*I*_ negative trends for South Africa and Mozambique over the last years were excluded from *R*_*I*_-functions and analyses. The first period of TB growth for Tanzania was excluded to properly show the trends of acceleration and decline in *R*_*I*_. In Brazil, there is a clear cloud of data around zero, suggesting an underlying diminishing returns process between *R*_*I*_ and TB cases.

Model fits revealed that TB dynamics was always dominated by a first order negative correlation between *R*_*I*_ and population size, which was captured by the discrete intra-specific competition model (Figs 1 and 2, S1 and S2 Figs, Table 1, S1 Table).

**Table 1:** TB model parameters for South Africa, Nigeria, Kenya, Mozambique, UR Tanzania, Zimbabwe, Brazil, Bangladesh, Cambodia and China.

		Parameters								
Models for TB dynamics			Rmax	K	Q	g	E	Lag	R^2^	AICs
South Africa										
1974–1987		•	0.1	**61366.44**	**4.862**				**0.691**	
1988–1993		•	0.279	**84513.45**	4.448				**0.89**	
1994–1999		•	0.391	**104840**	**2.407**				**0.958**	
2000–2012		•	0.37	**318300**	**1.562**				**0.818**	-21.897
2000–2012 HIV Lateral		•	0.372	**129800**	**5.424**	**0.036**		4	**0.931**	-29.439
2000–2012 HIV Vertical		•	5.7	**97890**	0.185	0.00000004		4	**0.95**	-31.837
Nigeria										
1974–1994		•	0.372	**13900**	**1.105**				**0.454**	
1994–2012		•	0.24	**90320**	1.264				**0.479**	-7.583
1994–2012 HIV Lateral		•	0.24	**30410**	6.624	**0.0034**		12	0.405	-17.531
Kenya										
1991–2012		•	0.181	**105700**	**4.226**				**0.825**	-37.913
1991–2012 HIV Lateral		•	0.181	**55870**	**4.511**	**0.0338**		8	**0.85**	-40.382
1991–2012 HIV Vertical		•	0.274	**47860**	**1.39**	**0.0000012**		**9**	**0.864**	-40.75
1991–2012 HIV Non-linear		•	0.187	**110600**	**3.402**	-0.00002		4	**0.861**	-40.447
Mozambique										
1974–1984		•	0.62	**6427**	**1.682**				**0.826**	
1985–1991		•	0.34	**15890**	**3.592**				**0.985**	
1991–2000		•	0.09	**25929**	1.859				0.504	
2001–2012		•	0.14	**48660**	**1.927**				**0.729**	-42.134
2001–2012 HIV Lateral		•	0.14	**24680**	**12.62**	**0.0169**		8	**0.926**	-53.34
UR Tanzania										
1976–1984		•	3.179	4977	0.162				0.45	
1982–1993		°	0.15	**59517**			**10490**		**0.679**	
1994–2007		•	0.121	**60688**	**4.211**				**0.697**	-47.121
1994–2007 HIV Lateral		•	0.121	31770	5.582	0.003		4	**0.832**	-45.795
Zimbabwe										
1974–1989		•	0.11	**5098**	1.283				0.316	
1989–2004		•	**0.314**	**54060**	**1.541**				**0.91**	-40.523
1989–2004 HIV Lateral		•	0.314	8906	**1.825**	**0.0249**		0	**0.833**	-42.521
1989–2004 HIV Vertical		•	0.981	11230	**0.289**	0.00000031		0	**0.84**	-38.612
1989–2004 HIV Non-linear		•	**0.226**	**54470**	53.95	-0.000027		0	**0.767**	-38.612
Brazil										
1974–1978										
1979–2006		•	0.28	**83010**	**3.159**				0.31	-66.785
1979–2006 HIV Lateral		•	0.28	**85720**	**3.09**	-0.0006		0	0.331	-66.235
Bangladesh										
1975–1991		•	1.38	**44220**	**0.71**				**0.873**	
1992–1999		•	0.54	**70930**	1.157				**0.725**	
1999–2004		°	0.224	**158236**			**46965**		**0.863**	
2004–2012		•	0.224	**151600**	**5.461**				**0.911**	-24.363
2004–2012 HIV Lateral		•	0.224	**125700**	**7.655**	**6.109**		7	0.564	-28.303
Cambodia										
1982–1990		•	0.227	**9995.11**	11.25				**0.69**	
1991–1997		•	0.39	**15182.04**	**4.924**				**0.902**	
2001–2012		•	0.249	**38350**	**2.316**				**0.728**	-35.648
2001–2012 HIV Lateral		•	0.249	**28520**	**5.561**	**0.0959**		10	**0.68**	-40.143
China										
1983–1993		•	0.328	**341000**	**2.041**				**0.794**	
1994–2001		•	0.349	**474200**	**3.273**				**0.837**	
2002–2012		•	0.286	**908900**	**3.646**				**0.95**	-32.806
2002–2012 HIV Lateral		•	0.286	**973900**	**3.791**	**-0.292**		11	**0.992**	-48.162

We also found other behaviors including *R*_*I*_ increase trend (e.g. UR Tanzania and Bangladesh), exponential growth (e.g. Mozambique), and circular *R*_*I*_ behavior (e.g. Myanmar and Nigeria), which suggest a predator prey interaction (S1 and S2 Figs, S1 Table). These behaviors were mostly restricted to the initial phases of TB time series, while the last periods were dominated by the logistic growth (Fig 2, S2 Fig, S1 Table). Myanmar and perhaps Nigeria showed the most drastic changes in *R*_*I*_ behavior (Figs 1 and 2, Table 1, S1 and S2 Figs, S1 Table). They started with a circular *R*_*I*_ behavior, while in the last years these countries displayed a logistic growth pattern. Curiously, both circular behaviors had a lag of approximately 7 years.

*R*_max_, *K*, *Q*, *E* and *g* are the logistic models parameters as in the text. R^2^ is the coefficient of determination which measures the endogenous component for TB dynamics. AICs is the Akaike Information Criteria. Lag refers to the year in which TB+HIV AICs showed the lowest value. *R*_max_ was estimated directly from the data and fixed, except for Vertical and Non-linear models in which it was estimated by nls regression. Significant results (p<0.05) are in boldfaces. For most of all countries, the TB growth periods were captured by the logistic model, denoted by “•”. The exceptions were UR Tanzania (1982–1993) and Bangladesh (1999–2004), which displayed an *R*_*I*_ increase with TB population size (the Allee model), denoted by “°”. See S1 Table for the results of other countries.

Since 1974, TB HBCs displayed 2 to 4 periods of logistic growth (Figs 1 and 2, S1 and S2 Figs). The coefficient of determination (R^2^) measures the endogenous component of a dynamic (i.e. how much *R*_*I*_ is determined by population size). In most periods of logistic growth, R^2^ was high suggesting that TB dynamics had always a strong endogenous component (Table 1 and S1 Table). In Africa, the average increase in *K* between the first and the last TB logistic growth periods was of 878%. Outside Africa it was of only 45.8%. *R*_max_ increased 21.9% in Africa, whereas it decreased 173.2% outside Africa. These general trends suggest an overall enhancement of the carrying capacity and an usually negative change in the maximum per capita rate of change, *R*_max_ (Table 2).

**Table 2:** Percent changes in *R*_max_ and *K* between the first and last TB logistic periods, ordered by country from the highest to the lowest changes in K.

Country	Region	Δ*R*_max_ (%)	Δ*K* (%)
Uganda	Africa	-86.1	2850.0
Zimbabwe	Africa	185.5	960.4
Kenya	Africa	-39.7	817.5
Mozambique	Africa	-77.4	657.1
Nigeria	Africa	55.6	434.7
South Africa	Africa	270.0	418.7
Ethiopia	Africa	44.4	368.2
RD Congo	Africa	-59.7	276.2
Myanmar	Asia	29.0	90.5
Cambodia	Asia	8.8	73.9
Thailand	Asia	-89.2	73.7
Bangladesh	Asia	-516.1	70.8
Pakistan	Asia	-243.1	70.5
China	Asia	-14.7	62.5
Viet Nam	Asia	0.0	50.0
India	Asia	-733.3	32.0
Philippines	Asia	-21.5	-17.1
Afghanistan	Asia	-151.7	-48.9

Introducing HIV as an exogenous variable in the last TB logistic period resulted in more parsimonious models than TB alone, based on AICs values (Table 1, S1 Table), principally in the African region. In general, R^2^ did not improve after including HIV into the TB model (Table 1, S1 Table), which can be explained by TB strong endogenous component. We also found some unexpected results. For example, based only in AICs results, in Uganda and Tanzania the lateral TB+HIV model was less parsimonious than TB model alone (Table 1, S1 Table), which can be explained by HIV shorter time series (not showed here). We were not able to test for time-lags greater than five years, which might have resulted in better AICs for these countries, where the link between HIV and TB is well known [7]. On the other hand, the TB+HIV model improved R^2^ in about 20% in both Uganda and Tanzania (S3 Text) supporting the inclusion of HIV lateral effect on TB logistic model.

Outside Africa, the TB+HIV models did not improve R^2^, neither decreased AICs, as observed for Bangladesh, Pakistan, Philippines and Vietnam (Table 1, S1 Table). For China, including HIV produced higher R^2^ and lower AIC than TB model alone. Yet, it produced a negative relationship (*g*, the HIV coefficient) between HIV levels and TB carrying capacity (*K*). We suggest that the statistical link between these diseases in China should be taken with caution. Overall, DT (detection rate), TS (treatment success), BCG coverage (Bacillus Calmette-Guérin vaccine) and per capita GDP (as a proxy of poverty) had a poor explanation power regarding TB and TB+HIV (lateral) model residuals (S2 Table).

## Discussion

Two major findings deserve to be highlighted: (i) besides some exceptions, since 1974, TB dynamics presented a strong endogenous component, a first order feedback between the TB population size and its population per capita rate of change; and (ii) TB models with HIV exogenous effects were more parsimonious than TB model alone, especially in the African region, These findings suggest a strong endogenous component and supports the known HIV effects on TB natural-history and dynamics.

We found that the simple logistic discrete model (for the infectious class) can capture the TB behavior for a particular period of time. Although the *R*_*I*_ decline trend may be also the result of improvements in health programs and life standards, the sequential logistic periods, the R^2^ levels between *R*_*I*_ and *I*_*t* − 1_, together with the weak correlations between non-HIV variables and model residuals, suggest that the endogenous view should be considered as a plausible hypothesis to explain and model TB infectious class dynamics.

The sequence of TB logistic periods of growth suggests a succession of population thresholds levels, from which TB is freed by its exogenous control strategies. Hence, the sequence of logistic growth patterns can be interpreted as a series of density-dependency chronological domains. Since 1974, TB has suffered changes on its equilibrium surface, changing only the parameter values (principally *K*), not the biological process underpinning its dynamics (i.e. diminishing returns, self-limiting). This resembles the fifth principle of population dynamics suggested by Berryman [23, 25], which is based on the “Minimum law” of Leibig. At a particular period of time, the population (i.e. the infectious class), at equilibrium, is limited by the self-limitting process for the lower resource available in the environment (i.e. susceptible class). During the last decades, HIV appearance not only represented a new TB period, but it has also been continuously pushing TB model parameters to a new globally stable landscape. Oncoming HIV and TB vaccines, in addition to new prophylaxes, combined with control strategies will probably change TB dynamic properties again, leading to a new period of a global decline [3–7].

The changes between the first and last TB model (Table 2) and the results of our TB+HIV models suggest that HIV probably acted as an exogenous forcing on TB dynamics. These results are in agreement with the expected HIV effects on TB dynamics. HIV simplifies the complex TB natural history. As HIV load increases, individuals are more susceptible to be infected by TB. When individuals are co-infected, HIV accelerates the progression rates from TB exposed to infectious stage. It has been estimated that one third of the global population is in the Exposed stage of TB [3], representing a large pool of individuals that can rapidly progress to active TB [9–13, 19, 20]. Yet, a co-infected individual is not necessarily more contagious than a HIV negative individual with active TB. These known HIV effects on TB may explain the disproportional *K* changes compared to *R*_max_ inside Africa, where the level of co-infection may reach 75% of TB cases [3].

Despite the main effect of HIV and the lack of statistical relationship between residuals and non-HIV variables, we cannot rule completely out the importance of these variables. Both TB and HIV are related to poverty and the other variables (DT, TS and BCG) may be correlated to poverty levels through governmental funding [33–40] (S4 Text). Almost all countries analyzed here were until recently European colonies. They share low per capita income levels, have deficient health infrastructures and suffer from internal political instabilities, which create a perfect environment to the spread of HIV. Also, most of the countries have suffered from armed conflicts, which may increase disease burdens due to their impacts on health services, food production and distribution, migrations and refugees overcrowding [41,42]. For example, the TB initial peaks in Afghanistan and in Viet Nam may be correlated to the war conflicts (S1 and S2 Figs).

TB reported cases may not exactly measure TB infected class and may be also a function of changes in diagnosis, reporting practices and case findings [12]. Until the spread of HIV, some positive trends could be explained by improvements in detection rate and not by TB threshold levels and the endogenous process, as suggested above. Despite these drawbacks, we used TB reported cases as a proxy of TB burden, which allowed describing empirically and theoretically TB time series since 1974, and we were also able to capture the known regionally HIV effect on TB burden in Africa. A finer scale analysis would reveal details about the contribution of case finding and reporting practices vs TB endogenous behavior to the periods prior the HIV spread. A finer scale analyses may also describe more complex scenarios and the effects of other variables including MDR-TB strains, HIV strains, the role of susceptible, exposed and recovered pools, urbanization process, ART availability, BCG waning immunity with human increase life expectancy, colinearities between DT and TS, among others [12–18, 43]. A finer scale analysis was not the scope of this study though. Our goal was to apply population ecology theory to explore TB trends and estimate HIV effects. We found distinct TB density-dependency domains captured by the logistic discrete model, and we found that HIV had an exogenous effect on TB carrying capacity, principally in Africa. The framework is based on the realized per capita population rate of change (*R*_*I*_), which is surrounded by plausible ecological principles, and hence, a good starting point to explore TB dynamics. Any country interested in its TB epidemic may use our results as hypotheses and move forward. For example, outside Africa, governments can substitute HIV lateral effect for DT, TS, BCG or per capita GDP, compare and select the best model based on statistical results and their knowledge about TB epidemic. In addition, it is also possible to test the endogenous and exogenous contributions at distinct scales inside the country (Regions, cities, etc), principally for large territory countries like Brazil. Any government may disentangle *R*_*I*_ in its components (new per capita infections and per capita mortality), explore which of them are more important for *R*_*I*_ trends and also explore the contributions of the endogenous and exogenous processes.

This study suggests that governments’ efforts in controlling TB epidemic were not enough to avoid the impact of HIV in TB, principally in African region, in agreement with many other studies and reports [3,4]. This does not mean that control policies are completely inefficient, since many TB related deaths are annually avoided by improvements in health care and the impact of HIV would be even greater than observed. This study also shows that governments can use simple tools to explore and identify the mechanisms underlying TB dynamics and other diseases. Hence, we suggest that this framework could be included as a supplement into WHO reports with minimal cost and time demanding efforts, which could provide insights, hypotheses and may allow testing and estimating the relationship between TB and other variables.

## Supporting Information

S1 Fig The HBCs time series not presented in the main text. (JPG)

S2 Fig The R-function of the HBCs time series not presented in the main text. (JPG)

S1 Table Statistical model results for the Ethiopia, Nigeria, DR Congo, Uganda, India, Afghanistan, Indonesia, Myanmar, Philippines, Thailand and Viet Nam. (DOC)

S2 Table Pearson´s product moment correlation coefficient between TB alone, TB+HIV lateral models`residuals and non-HIV variables. (DOC)

S1 Text TB natural-history, SEIR models and interactions between TB infectious and susceptible classes. (DOC)

S2 Text The cut-points of TB time series since 1974. (DOC)

S3 Text Degrees of freedom and noise of TB time series. (DOC)

S4 Text Poverty, TB and HIV. (DOC)
