# Supplementary figures and images for: Tuberculosis Epidemiology at the Country Scale: Self-Limiting Process and the HIV Effects
Source: PLoS One. 2016 Apr 19;11(4):e0153710. doi: 10.1371/journal.pone.0153710 (PMC4836699; doi:10.1371/journal.pone.0153710)

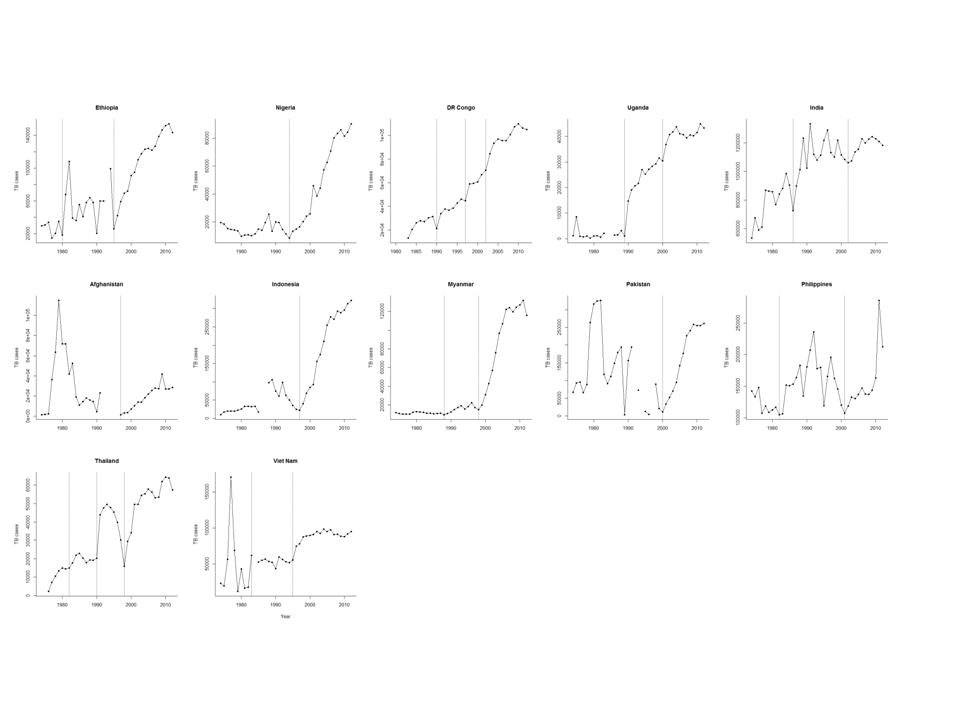

Supplement: S1 Fig — (JPG) [file pone.0153710.s001.jpg]

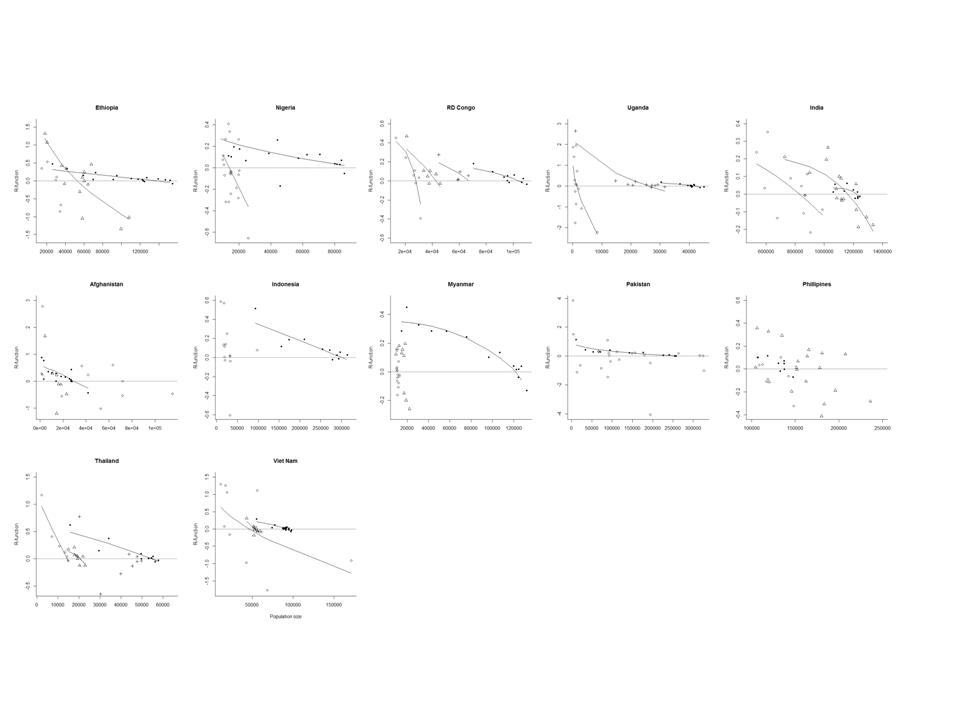

Supplement: S2 Fig — (JPG) [file pone.0153710.s002.jpg]
