# Supplementary material for: Tuberculosis Epidemiology at the Country Scale: Self-Limiting Process and the HIV Effects
Source: PLoS One. 2016 Apr 19;11(4):e0153710. doi: 10.1371/journal.pone.0153710 (PMC4836699; doi:10.1371/journal.pone.0153710)
Supplement: S1 Table — (DOC) [file pone.0153710.s003.doc]

Table S1. Statistical model results for the Ethiopia, Nigeria, DR Congo, Uganda, India, Afghanistan, Indonesia, Myanmar, Philippines, Thailand and Viet Nam.

|  |  |  |  |  |  |  |  |  |  |  |
| --- | --- | --- | --- | --- | --- | --- | --- | --- | --- | --- |
|  |  |  |  |  |  |  |  |  |  |  |
|  |  |  | Parameters |  |  |  |  |  |  |  |
|  |  |  |  |  |  |  |  |  |  |  |
| Models for TB dynamics | |  | Rmax | K | Q | g | E | Lag | R^2^ | AICs |
|  |  |  |  |  |  |  |  |  |  |  |
| Ethiopia |  |  |  |  |  |  |  |  |  |  |
|  |  |  |  |  |  |  |  |  |  |  |
| 1974-1979 |  | • | 0.277 | **29926** | 8.645 |  |  |  | **0.94** |  |
| 1980-1994 |  | • | 3.94 | **52600** | **0.333** |  |  |  | **0.798** |  |
| 1995-2012 |  | • | 0.4 | **140100** | **0.911** |  |  |  | **0.85** | -35.418 |
| 1995-2012 HIV Lateral | | • | 0.4 | **63570** | **2.357** | **0.086** |  | 7 | **0.908** | -40.126 |
| 1995-2012 HIV Vertical | | • | 4.54 | **70460** | 0.108 | 0.00000008 |  | 7 | **0.881** | -38.612 |
|  |  |  |  |  |  |  |  |  |  |  |
| Nigeria |  |  |  |  |  |  |  |  |  |  |
|  |  |  |  |  |  |  |  |  |  |  |
| 1974-1994 |  | • | 0.372 | **13900** | **1.105** |  |  |  | **0.454** |  |
| 1994-2012 |  | • | 0.24 | **90320** | 1.264 |  |  |  | **0.479** | -7.583 |
| 1994-2012 HIV Lateral | | • | 0.24 | **30410** | 6.624 | **0.0034** |  | 12 | 0.405 | -17.531 |
|  |  |  |  |  |  |  |  |  |  |  |
| DR Congo |  |  |  |  |  |  |  |  |  |  |
|  |  |  |  |  |  |  |  |  |  |  |
| 1983-1989 |  | • | 0.449 | **27907** | **3.725** |  |  |  | **0.905** |  |
| 1990-1996 |  | • | 0.469 | **42770** | 1.748 |  |  |  | **0.86** |  |
| 1997-2001 |  | • | 0.274 | **66959** | 3 |  |  |  | **0.765** |  |
| 2002-2012 |  | • | 0.181 | **105000** | **3.186** |  |  |  | **0.838** | -33.55 |
| 2002-2012 HIV Lateral | | • | 0.181 | 15620 | **6.416** | **0.189** |  | 4 | **0.886** | -34.736 |
|  | | |  |  |  |  |  |  |  |  |
| Uganda |  |  |  |  |  |  |  |  |  |  |
|  |  |  |  |  |  |  |  |  |  |  |
| 1974-1988 |  | • | 1.38 | **1428** | **0.554** |  |  |  | **0.634** |  |
| 1989-1999 |  | • | 2.64 | **26290** | **0.481** |  |  |  | **0.671** |  |
| 2000-2012 |  | • | 0.192 | **42126** | **4.447** |  |  |  | **0.636** | -39.527 |
| 2000-2012 HIV Lateral | | • | 0.192 | **41920** | **4.418** | 0.0026 |  | 3 | **0.845** | -37.531 |
|  |  |  |  |  |  |  |  |  |  |  |
| India |  |  |  |  |  |  |  |  |  |  |
|  |  |  |  |  |  |  |  |  |  |  |
| 1974-1986 |  | • | 0.3 | **829600** | 1.931 |  |  |  | **0.592** |  |
| 1987-2002 |  | • | 0.22 | **1163000** | **4.842** |  |  |  | **0.82** |  |
| 2002-2012 |  | • | 0.036 | **1220000** | 25.68 |  |  |  | **0.71** | -43.42 |
| 2002-2012 HIV Lateral | | • | 0.036 | **784300** | 35.5 | **0.166** |  | 6 | **0.824** | -44.854 |
|  |  |  |  |  |  |  |  |  |  |  |
|  |  |  |  |  |  |  |  |  |  |  |
| Afghanistan |  |  |  |  |  |  |  |  |  |  |
|  |  |  |  |  |  |  |  |  |  |  |
| 1974-1984 |  | • | 2.19 | 41820 | 0.182 |  |  |  | 0.53 |  |
| 1985-1991 |  |  |  |  |  |  |  |  |  |  |
| 1997-2012 |  | • | 0.87 | **28080** | **0.447** |  |  |  | 0.492 | 1.286 |
| 1997-2012 HIV Lateral | | • | 0.87 | 8339 | 0.617 | 6.59 |  | 0 | 0.491 | 2.861 |
|  |  |  |  |  |  |  |  |  |  |  |
| Indonesia |  |  |  |  |  |  |  |  |  |  |
|  |  |  |  |  |  |  |  |  |  |  |
| 1974-1984 |  |  |  |  |  |  |  |  |  |  |
| 1984-1998 |  |  |  |  |  |  |  |  |  |  |
| 1998-2002 |  | • | 0.6 | **89070** | **2.227** |  |  |  | **0.985** |  |
| 2002-2012 |  | • | 0.514 | 303300 | 1 |  |  |  | **0.872** | -21 |
| 2002-2012 HIV Lateral | | • | 0.514 | **211400** | **1.617** | **0.207** |  | 0 | **0.903** | -21.876 |
|  |  |  |  |  |  |  |  |  |  |  |
| Myanmar |  |  |  |  |  |  |  |  |  |  |
|  |  |  |  |  |  |  |  |  |  |  |
| 1974-1987 |  | • | 0.25 | **11693.2** | **7.249** |  |  |  | 0.323 |  |
| 1974-1987 |  | • | 0.194 | **11746** | **9.417** |  |  |  | **0.95** |  |
| 1988-1996 |  | • | 0.149 | **18301** | **5.485** |  |  |  | **0.763** |  |
| 1997-2012 |  | • | **0.352** | **123500** | **2.258** |  |  |  | **0.955** | -37.588 |
| 1997-2012 HIV Lateral | | • | 0.352 | 5888 | **3.768** | **0.41** |  | 7 | **0.963** | -40.373 |
| 1997-2012 HIV Vertical | | • | 0.152 | 6.855 | 0.64 | 0.0000188 |  | 6 | **0.962** | -37.859 |
| 1997-2012 HIV Non-linear | | • | **0.355** | **125300** | 17.83 | 0.0000541 |  | 2 | **0.962** | -37.964 |
|  |  |  |  |  |  |  |  |  |  |  |
| Pakistan |  |  |  |  |  |  |  |  |  |  |
|  |  |  |  |  |  |  |  |  |  |  |
| 1974-1999 |  | • | 3.86 | 76870 | 0.135 |  |  |  | **0.546** |  |
| 2000-2012 |  | • | 1.125 | **261000** | **0.362** |  |  |  | **0.89** | -7.566 |
| 2000-2012 HIV Lateral | | • | 1.125 | 210200 | **0.399** | 1.299 |  | 2 | **0.892** | -5.763 |
|  |  |  |  |  |  |  |  |  |  |  |
| Philippines |  |  |  |  |  |  |  |  |  |  |
|  |  |  |  |  |  |  |  |  |  |  |
| 1974-1981 |  |  |  |  |  |  |  |  |  |  |
| 1982-1999 |  | • | 0.13 | **164400** | 3.096 |  |  |  | -0.415 |  |
| 2000-2008 |  | • | 0.107 | **140400** | **9.83** |  |  |  | 0.073 | -26.319 |
| 2000-2008 HIV Lateral | | • | 0.107 | **113700** | **11.91** | 4.421 |  | 3 | -0.186 | -25.507 |
|  |  |  |  |  |  |  |  |  |  |  |
| Thailand |  |  |  |  |  |  |  |  |  |  |
|  |  |  |  |  |  |  |  |  |  |  |
| 1976-1981 |  | • | 1.169 | **14350** | 0.921 |  |  |  | **0.965** |  |
| 1982-1989 |  | • | 0.171 | **20723.5** | 4.823 |  |  |  | **0.73** |  |
| 1990-1997 |  |  |  |  |  |  |  |  |  |  |
| 1998-2007 |  | • | 0.618 | **54540** | **1.274** |  |  |  | **0.911** | -15.096 |
| 1998-2007 HIV Lateral | | • | 0.618 | 12290 | **3.046** | **0.06** |  | 8 | **0.944** | -17.39 |
|  |  |  |  |  |  |  |  |  |  |  |
| Viet Nam |  |  |  |  |  |  |  |  |  |  |
|  |  |  |  |  |  |  |  |  |  |  |
| 1974-1983 |  | • | 1.179 | 46310 | 0.561 |  |  |  | **0.595** |  |
| 1985-1993 |  | • | 0.31 | **5413** | 3.367 |  |  |  | **0.746** |  |
| 1995-2012 |  | • | 0.29 | **92580** | **2.518** |  |  |  | **0.865** | -55.86 |
| 1995-2012 HIV Lateral | | • | 0.29 | **92190** | **2.541** | 0.0019 |  | 1 | **0.865** | -53.876 |
|  |  |  |  |  |  |  |  |  |  |  |
|  |  |  |  |  |  |  |  |  |  |  |

$R\text{max}$,$K$, $Q$, $E$and $g$ are the logistic models parameters as in the text. R^2^ is the coefficient of determination which measures the endogenous component for TB dynamics. AICs is the Akaike Information Criteria. Lag refers to the year in which TB+HIV AICs showed the lowest value. $R\text{max}$ was estimated directly from the data and fixed, except for Vertical and Non-linear models in which it was estimated by nls regression. Most of all countries TB growth periods were captured by the logistic model. The exceptions were: Nigeria (1974-1994), Myanmar (1974-1987), both with initial R circular behavior (logistic growth with TB time-lag of 7 years); Thailand (1990-1997), Afghanistan (1985-1991), both with a TB pick; and, Indonesia (1999-2004) and Philippines (1974-1981) with TB decline trend. Significant results (p<0.05) are in boldfaces.
