# Supplementary material for: Tuberculosis Epidemiology at the Country Scale: Self-Limiting Process and the HIV Effects
Source: PLoS One. 2016 Apr 19;11(4):e0153710. doi: 10.1371/journal.pone.0153710 (PMC4836699; doi:10.1371/journal.pone.0153710)
Supplement: S2 Table — (DOC) [file pone.0153710.s004.doc]

Table S2. Pearson´s product moment correlation coefficient between TB alone, TB+HIV lateral models` residuals and non-HIV variables.

Significant results are in boldfaces. p (df) refers to p-values and degrees of freedom. Only the results for more than 5 degrees of freedom are showed.

|  |  |  |  |  |  |  |  |  |
| --- | --- | --- | --- | --- | --- | --- | --- | --- |
|  |  |  |  |  |  |  |  |  |
| Country | DT | p (df) | TS | p (df) | BCG | p (df) | GDP | p (df) |
|  |  |  |  |  |  |  |  |  |
| Brazil |  |  |  |  |  |  |  |  |
|  |  |  |  |  |  |  |  |  |
| 1979-2006 | -0.429 | 0.109 (13) | -0.329 | 0.295 (10) | -0.132 | 0.517 (24) | **-0.484** | **0.01 (25)** |
|  |  |  |  |  |  |  |  |  |
| Bangladesh |  |  |  |  |  |  |  |  |
|  |  |  |  |  |  |  |  |  |
| 1975-1991 |  |  |  |  | -0.096 | 0.777 (9) | 0.002 | 0.991 (14) |
| 1992-1999 | -0.228 | 0.586 (6) |  |  | 0.115 | 0.786 (6) | 0.194 | 0.644 (6) |
| 2004-2012 |  |  |  |  |  |  | 0.287 | 0.489 (6) |
| 2004-2012 HIV |  |  |  |  |  |  | -0.206 | 0.623 (6) |
|  |  |  |  |  |  |  |  |  |
| Cambodia |  |  |  |  |  |  |  |  |
|  |  |  |  |  |  |  |  |  |
| 1991-1997 | -0.314 | 0.492 (5) |  |  | 0.121 | 0.796 (5) |  |  |
| 2001-2012 | 0.126 | 0.745 (7) | 0.326 | 0.391 (7) | -0.227 | 0.588 (6) | 0.031 | 0.927 (9) |
| 2001-2012 HIV | 0.2 | 0.604 (7) | 0.155 | 0.688 (7) | -0.089 | 0.834 (6) | -0.13 | 0.702 (9) |
|  |  |  |  |  |  |  |  |  |
| China |  |  |  |  |  |  |  |  |
|  |  |  |  |  |  |  |  |  |
| 1983-1993 |  |  |  |  |  |  | 0.159 | 0.638 (9) |
| 1994-2001 | -0.62 | 0.1 (6) | **-0.92** | **0.001 (6)** | 0.003 | 0.992 (6) | -0.701 | 0.052 (6) |
| 2002-2012 | -0.456 | 0.216 (7) | -0.578 | 0.132 (6) | -0.498 | 0.208 (6) | **-0.832** | **0.002 (8)** |
| 2002-2012 HIV | -0.368 | 0.329 (7) | -0.648 | 0.081 (6) | 0.0001 | 0.999 (6) | -0.165 | 0.646 (8) |
|  |  |  |  |  |  |  |  |  |
| India |  |  |  |  |  |  |  |  |
|  |  |  |  |  |  |  |  |  |
| 1974-1986 |  |  |  |  |  |  | 0.057 | 0.859 (10) |
| 1987-2002 |  |  |  |  | 0.159 | 0.554 (14) | -0.266 | 0.319 (14) |
| 2000-2012 | 0.12 | 0.757 (7) | 0.494 | 0.213 (6) | 0.419 | 0.348 (5) | -0.186 | 0.606 (8) |
| 2002-2012 HIV | -0.054 | 0.889 (7) | 0.015 | 0.971 (6) | -0.094 | 0.839 (5) | -0.217 | 0.545 (8) |
|  |  |  |  |  |  |  |  |  |
| Apheganistan |  |  |  |  |  |  |  |  |
|  |  |  |  |  |  |  |  |  |
| 1997-2012 | 0.15 | 0.623 (11) | -0.277 | 0.382 (10) | 0.225 | 0.48 (10) | 0.096 | 0.777 (9) |
| 1997-2012 HIV | 0.228 | 0.452 (11) | -0.209 | 0.513 (10) | 0.296 | 0.349 (10) | -0.179 | 0.596 (9) |
|  |  |  |  |  |  |  |  |  |
|  |  |  |  |  |  |  |  |  |
| Indonesia |  |  |  |  |  |  |  |  |
|  |  |  |  |  |  |  |  |  |
| 1974-1984 |  |  |  |  | 0.743 | 0.055 (5) | -0.124 | 0.686 (11) |
| 2001-2012 | -0.236 | 0.51 (8) | -0.05 | 0.897 (7) | -0.108 | 0.782 (7) | 0.125 | 0.712 (9) |
| 2001-2012 HIV | -0.141 | 0.697 (8) | 0.155 | 0.69 (7) | 0.031 | 0.936 (7) | -0.093 | 0.785 (9) |
|  |  |  |  |  |  |  |  |  |
|  |  |  |  |  |  |  |  |  |
| Myanmar |  |  |  |  |  |  |  |  |
|  |  |  |  |  |  |  |  |  |
| 1997-2012 | 0.15 | 0.623 (11) | -0.058 | 0.856 (10) | -0.82 | 0.797 (10) |  |  |
| 1997-2012 HIV | -0.016 | 0.958 (11) | -0.272 | 0.391 (10) | -0.272 | 0.392 (10) |  |  |
|  |  |  |  |  |  |  |  |  |
| Pakisthan |  |  |  |  |  |  |  |  |
|  |  |  |  |  |  |  |  |  |
| 2000-2012 | 0.004 | 0.988 (9) | -0.018 | 0.959 (8) | 0.433 | 0.211 (8) | 0.101 | 0.754 (10) |
| 2000-2012 HIV | 0.02 | 0.951 (9) | 0.025 | 0.944 (8) | 0.401 | 0.249 (8) | 0.089 | 0.783 (10) |
|  |  |  |  |  |  |  |  |  |
| Philipines |  |  |  |  |  |  |  |  |
|  |  |  |  |  |  |  |  |  |
| 2000-2008 | 0.478 | 0.23 (6) | -0.122 | 0.773 (6) | -0.057 | 0.892 (6) | 0.196 | 0.641 (6) |
| 2000-2008 HIV | 0.176 | 0.676 (6) | -0.309 | 0.455 (6) | -0.235 | 0.575 (6) | -0.156 | 0.711 (6) |
|  |  |  |  |  |  |  |  |  |
| Thailand |  |  |  |  |  |  |  |  |
|  |  |  |  |  |  |  |  |  |
| 1982-1989 |  |  |  |  | 0.181 | 0.667 (6) | -0.382 | 0.349 (6) |
| 1998-2007 | -0.623 | 0.053 (8) |  |  | **0.806** | **0.008 (7)** | -0.024 | 0.947 (8) |
| 1998-2007 HIV | **-0.725** | **0.017 (8)** |  |  | **0.802** | **0.009 (7)** | -0.277 | 0.437 (8) |
|  |  |  |  |  |  |  |  |  |
| Vietnam |  |  |  |  |  |  |  |  |
|  |  |  |  |  |  |  |  |  |
| 1974-1983 |  |  |  |  | -0.256 | 0.473 (8) | 0.022 | 0.95 (8) |
| 1985-1993 |  |  |  |  | -0.172 | 0.633 (8) | 0.027 | 0.94 (8) |
| 1995-2012 |  |  | -0.082 | 0.769 (13) | **-0.747** | **0.005 (10)** | -0.03 | 0.907 (9) |
| 1995-2012 HIV | -0.344 | 0.191 (14) |  |  | 0.377 | 0.165 (13) | -0.085 | 0.743 (15) |
|  |  |  |  |  |  |  |  |  |
| South Africa |  |  |  |  |  |  |  |  |
|  |  |  |  |  |  |  |  |  |
| 1974-1987 |  |  |  |  |  |  | 0.319 | 0.265 (12) |
| 1988-1993 |  |  |  |  |  |  |  |  |
| 1994-1999 |  |  |  |  |  |  |  |  |
| 2000-2012 | 0.463 | 0.151 (9) | 0.179 | 0.597 (9) | -0.627 | 0.07 (7) | 0.204 | 0.545 (9) |
| 2000-2012 HIV | 0.044 | 0.896 (9) | -0.244 | 0.469 (9) | -0.089 | 0.818 (7) | -0.247 | 0.463 (9) |
|  |  |  |  |  |  |  |  |  |
| Kenya |  |  |  |  |  |  |  |  |
|  |  |  |  |  |  |  |  |  |
| 1978-1990 |  |  |  |  |  |  | **0.706** | **0.022 (8)** |
| 1991-2012 | -0.161 | 0.497 (18) | 0.09 | 0.72 (14) | -0.082 | 0.736 (17) | -0.068 | 0.767 (19) |
| 1991-2012 HIV | -0.2 | 0.395 (14) | -0.035 | 0.897 (14) | -0.101 | 0.68 (17) | -0.165 | 0.474 (19) |
|  |  |  |  |  |  |  |  |  |
| Mozambique |  |  |  |  |  |  |  |  |
|  |  |  |  |  |  |  |  |  |
| 1985-1991 |  |  |  |  | -0.05 | 0.913 (5) | 0.112 | 0.809 (5) |
| 1991-2000 | -0.104 | 0.788 (7) |  |  | 0.0009 | 0.998 (7) | 0.095 | 0.807 (7) |
| 2001-2012 | -0.469 | 0.17 (8) | -0.106 | 0.785 (7) | 0.154 | 0.692 (7) | 0.0002 | 0.993 (9) |
| 2001-2012 HIV | -0.155 | 0.667 (8) | 0.29 | 0.447 (7) | -0.247 | 0.521 (7) | -0.03 | 0.925 (9) |
|  |  |  |  |  |  |  |  |  |
| Zimbabwe |  |  |  |  |  |  |  |  |
|  |  |  |  |  |  |  |  |  |
| 1974-1983 |  |  |  |  |  |  | 0.099 | 0.798 (7) |
| 1984-1988 |  |  |  |  |  |  |  |  |
| 1989-2004 | 0.146 | 0.603 (13) | -0.021 | 0.951 (9) | -0.36 | 0.169 (14) | 0.37 | 0.157 (14) |
| 1989-2004 HIV | -0.117 | 0.69 (12) | 0.191 | 0.596 (8) | 0.139 | 0.62 (13) | -0.08 | 0.774 (13) |
| 2001-2012 HIV | -0.469 | 0.17 (8) | -0.106 | 0.785 (7) | 0.154 | 0.692 (7) | 0.0002 | 0.993 (9) |
|  |  |  |  |  |  |  |  |  |
| Nigeria |  |  |  |  |  |  |  |  |
|  |  |  |  |  |  |  |  |  |
| 1974-1993 |  |  |  |  | -0.031 | 0.927 (9) |  |  |
| 1994-1999 |  |  |  |  |  |  |  |  |
| 1999_2012 | -0.068 | 0.84 (9) | -0.08 | 0.821 (8) | **-0.858** | **0.006 (6)** |  |  |
| 1999_2012 HIV | -0.12 | 0.724 (9) | 0.002 | 0.994 (8) | **-0.78** | **0.022 (6)** |  |  |
|  |  |  |  |  |  |  |  |  |
| R. D. Congo |  |  |  |  |  |  |  |  |
|  |  |  |  |  |  |  |  |  |
| 1990-1996 | -0.477 | 0.279 (5) |  |  | 0.385 | 0.45 (4) | 0.115 | 0.805 (5) |
| 1997-2001 |  |  |  |  |  |  |  |  |
| 2002-2012 |  |  |  |  |  |  |  |  |
| 2002-2012 HIV | -0.324 | 0.395 (7) | -0.319 | 0.44 (6) | -0.38 | 0.352 (6) | -0.208 | 0.563 (8) |
|  |  |  |  |  |  |  |  |  |
| U. R. Tanzania |  |  |  |  |  |  |  |  |
|  |  |  |  |  |  |  |  |  |
| 1994-2007 | -0.187 | 0.538 (11) | 0.0272 | 0.929 (11) |  |  |  |  |
| 1994-2007 HIV | -0.256 | 0.396 (11) | -0.046 | 0.887 (11) |  |  | -0.193 | 0.526(11) |
|  |  |  |  |  |  |  |  |  |
| Uganda |  |  |  |  |  |  |  |  |
|  |  |  |  |  |  |  |  |  |
| 1989-1999 |  |  |  |  |  |  |  |  |
| 2000-2012 | 0.193 | 0.569 (9) |  |  | -0.348 | 0.293 (9) | 0.368 | 0.264 (9) |
| 2000-2012 HIV | -0.522 | 0.12 (8) | **-0.712** | **0.031 (7)** | 0.64 | 0.062 (7) | -0.051 | 0.873 (10) |
|  |  |  |  |  |  |  |  |  |
| Zimbabwe |  |  |  |  |  |  |  |  |
|  |  |  |  |  |  |  |  |  |
| 1974-1983 |  |  |  |  |  |  | 0.099 | 0.798 (7) |
| 1984-1988 |  |  |  |  |  |  |  |  |
| 1989-2004 | 0.146 | 0.603 (13) | -0.021 | 0.951 (9) | -0.36 | 0.169 (14) | 0.37 | 0.157 (14) |
| 1989-2004 HIV | -0.117 | 0.69 (12) | 0.191 | 0.596 (8) | 0.139 | 0.62 (13) | -0.08 | 0.774 (13) |
|  |  |  |  |  |  |  |  |  |
|  |  |  |  |  |  |  |  |  |
