# Supplementary material for: Tuberculosis Epidemiology at the Country Scale: Self-Limiting Process and the HIV Effects
Source: PLoS One. 2016 Apr 19;11(4):e0153710. doi: 10.1371/journal.pone.0153710 (PMC4836699; doi:10.1371/journal.pone.0153710)
Supplement: S1 Text — (DOC) [file pone.0153710.s005.doc]

S1. TB has a complex natural-history, and presents real challenges to model its dynamics. Individuals infected with TB may either never develop active TB, or they may develop it in a few months or years after contagion. Once they have developed TB, individuals can recover from it with or without medical help, or they may die from it. After recovery, individuals may be infected by the same or other strains. The complex TB natural-history would require a large amount of parameters to be estimated for deciphering TB dynamics at country scale following the SEIR approach. Hence, we deviated from the usual SEIR type models. Here we focused only on TB annually reported cases (e.g. diagnosed) aiming to reveal general TB dynamic patterns of the infected class at a country scale. The relationship between infected and susceptible individuals is usually assumed to be a predator-prey interaction or a metapopulation dynamics, with susceptible individuals representing vacant patches that can be occupied by pathogens. In both cases the dynamics of infected individuals (or occupied patches) in a large population is an interplay between the recruitment of new susceptible individuals (birth rate and vaccination coverage) and the transmission rate (i.e. how fast individuals are recruited to the infected class). Higher and lower birth rates lead, respectively, to a faster or lower replenishment of susceptible individuals. When the transmission rate is constant, a rapid replenishment leads to lower oscillation periods of the infected class and disease dynamics can be represented by a simple equilibrium point. Here, instead of starting with a predator-prey model as a default to model TB dynamics, we first looked at the data and then, decided when TB behaved as a first or a second order process.
