# Supplementary material for: Tuberculosis Epidemiology at the Country Scale: Self-Limiting Process and the HIV Effects
Source: PLoS One. 2016 Apr 19;11(4):e0153710. doi: 10.1371/journal.pone.0153710 (PMC4836699; doi:10.1371/journal.pone.0153710)
Supplement: S2 Text — (DOC) [file pone.0153710.s006.doc]

S2. It was a challenge to analyze data since 1974. TB notified cases time series are filled with noise and abrupt changes. Analyzing the entire time period (1974-2012) ignoring these changes would put together distinct equilibrium surface domains, leading to the loss of details and insights about TB trends and mechanisms. The size of the time series may influence the doubt related to where and how much to cut it. Longer time series capture more exogenous changes and probably more dynamic behaviors, increasing the uncertainty about where to cut them. We could have chosen to analyze only the last 15 or 20 years of TB. It might have produced a cleaner view of patterns. However, our goal was to describe TB dynamics as far we could get for the HBCs countries.

Data visual inspection is a common step used by modelers to get some clues about distinct chronological domains and mechanisms. Data inspection suggested that TB suffered categorical changes. The only way in dealing with this type of change is to cut the time series. For example, if was declining and suddenly increased and then started to decline again, we assumed that the time series should be cut prior to the increase and the periods should be analyzed separately, while keeping the global view of changes since 1974. We analyzed TB time series since 1974, but we focused in the last TB period, hence distinct cut-points would not affect the pattern of TB logistic growth in the last years and the effect of HIV on TB model parameters.
