# Supplementary material for: Tuberculosis Epidemiology at the Country Scale: Self-Limiting Process and the HIV Effects
Source: PLoS One. 2016 Apr 19;11(4):e0153710. doi: 10.1371/journal.pone.0153710 (PMC4836699; doi:10.1371/journal.pone.0153710)
Supplement: S3 Text — (DOC) [file pone.0153710.s007.doc]

S3. The fact that we found up to 4 periods of TB growth reduced the degrees of freedom to estimate the effects of TB endogenous component, HIV and other variables on TB dynamics. We found even more than 4 TB growth periods, but we used extended periods to get more degrees of freedom to capture the general pattern. For example, R.D. Congo and Ethiopia showed another TB logistic period from around 2005 until 2012.

TB time series are filled with noise. Noise may be the main reason for the poor fit of TB logistic models for Brazil, Bangladesh, Philippines and Tanzania. For example, in Brazil the logistic model explained only 30 % (non-significant) of observed behavior. Yet, there is a visually clear data cloud indicating a general decline trend with the number of TB infections for Brazil. Increasing the interval between observations should produce a better statistical result for the logistic pattern graphically observed. Despite some statistically non-significant results due to noise and outliers, we suggest that the logistic or cooperation models should be nevertheless considered as plausible hypotheses for explaining TB infected class dynamics.

The periods in which increased with TB population size (Tanzania and Bangladesh) may also be explained by continuous inflow of individuals from Exposed to Infected class as HIV spread. In this sense, TB levels may be simply following the HIV levels without any intra-population process. Another hypothesis is that the positive trend may be due to the urbanization process, which may have increased contact rate and the per capita rate of transmission. We believe that a specific HIV study following the framework adopted here is needed to decipher the endogenous and exogenous effects on its dynamics at country scale.
