# Supplementary material for: Tuberculosis Epidemiology at the Country Scale: Self-Limiting Process and the HIV Effects
Source: PLoS One. 2016 Apr 19;11(4):e0153710. doi: 10.1371/journal.pone.0153710 (PMC4836699; doi:10.1371/journal.pone.0153710)
Supplement: S4 Text — (DOC) [file pone.0153710.s008.doc]

S4. Undernourishment increases individual susceptibility to and progression rates of TB. Low household income levels may force individuals to engage on paid sex, increasing the probability to contract HIV. In poor countries, an individual may be more prompted to be infected and to develop a disease and less likely to recover from it. Sick individuals have their productivity reduced, decreasing the household and country incomes, and potential future funding to per capita natural quality, increasing susceptibility of individuals to diseases. For example, in the last years, some sub-Saharan countries have shown reductions on per capita GDP due to increased AIDS mortality, with TB being the main morbidity factor.
